# Diabetes-Independent Retinal Phenotypes in an Aldose Reductase Transgenic Mouse Model

**DOI:** 10.3390/metabo11070450

**Published:** 2021-07-10

**Authors:** Jonathan Mark Petrash, Biehuoy Shieh, David A. Ammar, Michelle G. Pedler, David J. Orlicky

**Affiliations:** 1 Department of Ophthalmology, University of Colorado School of Medicine, 12800 E. 19th Ave., Aurora, CO 80045, USA; Biehuoy.Shieh@CUAnschutz.edu (B.S.); Michelle.Pedler@CUAnschutz.edu (M.G.P.); 2 Department of Pharmaceutical Sciences, Skaggs School of Pharmacy and Pharmaceutical Sciences, University of Colorado Anschutz Medical Campus, 1635 Aurora Ct, Aurora, CO 80045, USA; 3 Lions Eye Institute for Transplant and Research, 1410 N 21st St, Tampa, FL 33605, USA; DAmmar@Lionseyeinstitute.org; 4 Department of Pathology, University of Colorado School of Medicine, 12800 E. 19th Ave., Aurora, CO 80045, USA; David.Orlicky@CUAnschutz.edu

**Keywords:** aldose reductase, Müller glia, retinal ganglion cell, Sorbinil, pattern electroretinogram, retinal microglia

## Abstract

Aldose reductase (AR), the first and rate-limiting enzyme of the polyol pathway, has been implicated in the onset and development of the ocular complications of diabetes, including cataracts and retinopathy. Despite decades of research conducted to address possible mechanisms, questions still persist in understanding if or how AR contributes to imbalances leading to diabetic eye disease. To address these questions, we created a strain of transgenic mice engineered for the overexpression of human AR (AR-Tg). In the course of monitoring these animals for age-related retinal phenotypes, we observed signs of Müller cell gliosis characterized by strong immunostaining for glial fibrillary acidic protein. In addition, we observed increased staining for Iba1, consistent with an increase in the number of retinal microglia, a marker of retinal inflammation. Compared to age-matched nontransgenic controls, AR-Tg mice showed an age-dependent loss of Brn3a-positive retinal ganglion cells and an associated decrease in PERG amplitude. Both RGC-related phenotypes were rescued in animals treated with Sorbinil in drinking water. These results support the hypothesis that increased levels of AR may be a risk factor for structural and functional changes known to accompany retinopathy in humans.

## 1. Introduction

Aldose reductase (AR) is an NADPH-dependent aldo-keto reductase that has been extensively studied as the first and rate-limiting enzyme of the polyol pathway [1]. The elevation of glucose levels, such as in diabetes mellitus, results in an increase in glucose flux through this pathway in diabetic target tissues, leading to an increased accumulation of pathway products, including sorbitol and fructose, as well as a disruption of the redox balances of nicotinamide cofactors NAD(P) and NAD(P)H [2]. In addition to catalytically facilitating these metabolic imbalances in diabetes, recent evidence suggests that AR may also be involved in a noncatalytic fashion in regulating inflammation and tissue remodeling [3,4,5]. To model the polyol pathway as a druggable target for diabetic cataracts, we created a mouse strain on the C57BL/6 background modified with the insertion of sequences encoding human AR (AKR1B1) under the transcriptional control of a hybrid lens crystallin gene promoter construct. As expected, high levels of AKR1B1 were found in the transgenic lens, giving rise to an elevated risk of diabetic cataracts [6]. In the course of further phenotyping this mouse strain, we noticed an increasingly prominent thickening of the tissue layer encompassing the retinal nerve fiber layer (RNFL) and the retinal ganglion cell layer (designated RNFL complex) in AR-Tg mice, beginning around 15 weeks of age. The purpose of this work was to characterize this phenotype and elucidate the possible consequences of this change in visual function in the mouse model. Immunostaining results showed that expression of the AR-Tg induced a proinflammatory state, as evidenced by the elevated expression of classical markers of retinal inflammation, including glial fibrillary acidic protein (GFAP), cellular retinaldehyde binding protein (CRALBP), increased abundance of activated retinal microglia, and loss of retinal ganglion cells. The levels of such indicators of inflammation were largely attenuated by the treatment of AR-Tg mice with Sorbinil, a well-characterized AR inhibitor. These results demonstrate that the activation of AR gene expression in the eye leads to structural and functional changes that translate into a vision-threatening inflammatory phenotype even in the absence of diabetes. 

## 2. Results

### 2.1. AR-Dependent Changes to Cells Associated with the Retinal Nerve Fiber Layer/Retinal Ganglion Cell Layer Complex in Ar-Transgenic Mice

**Figure 1:**
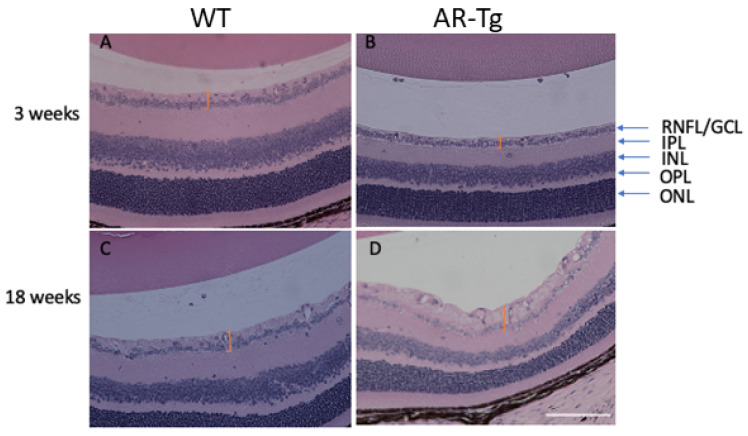
Age-dependent thickening of the retinal nerve fiber layer complex in AR-Tg mice. At 3 weeks of age, the retinas from WT mice (**A**) were qualitatively similar to retinas from AR-Tg mice (**B**). However, by 18 weeks of age, WT mouse retinas (**C**) were thinner at the nerve fiber layer/RGC layer as compared with AR-Tg retinas (**D**). Orange brackets demarcate the areas of comparison. Retinal layers are indicated with arrows: RNFL/GCL, retinal nuclear layer/ganglion cell layer complex; IPL, inner plexiform layer; INL, inner nuclear layer; OPL, outer plexiform layer; ONL, outer nuclear layer. Scale bar = 200 µm.

**Figure 2:**
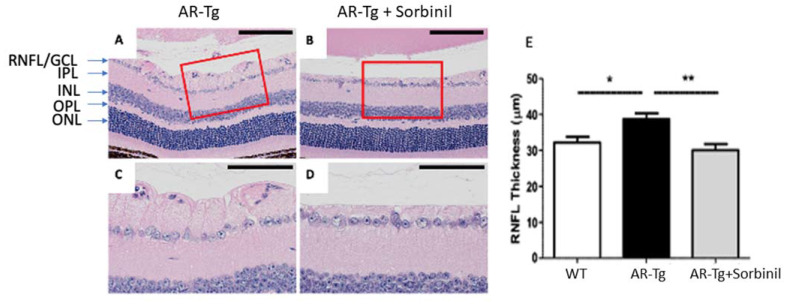
Sorbinil inhibition of retinal thickening in AR-Tg mice. Histological comparison of RNFL complex thickness of untreated AR-Tg controls (**A**) and Sorbinil-treated AR-Tg mice (**B**). Scale bar = 100 µm. Areas outlined in red are shown in expanded view in (**C**,**D**), where scale bar = 50 µm. Measurements of RNFL complex thickness (**E**) were carried out at 18 weeks of age, including ≥ 4 mice/observation group. H&E-stained histological sections shown are representative of six eyes. * *p* < 0.05; ** *p* < 0.01. Labeling of retinal layers is as in Figure 1.

We previously reported on a series of mutant mouse strains designed for the over-expression of human aldose reductase (AKR1B1) in the lens epithelium and the outer cortical fiber cells to support mechanistic studies of diabetic cataract formation [6,7]. Considering that we used lens crystallin gene promoters in the transgene construct, we observed the expected strong expression of AKR1B1 in the lens and an associated elevated risk for diabetes-dependent cataract formation [6]. In the course of phenotyping the mutant strain at different ages, we also observed in 18-week-old mice a marked thickening of the retinal nerve fiber layer and the retinal ganglion cell layer (RNFL complex), organized as a layer of cellular material adjacent to the retinal inner limiting membrane (Figure 1). In contrast, an increase in RNFL complex thickening was not apparent in the retinae from 18-week-old nontransgenic control mice or in AR-Tg or control mice at 3 weeks of age (Figure 1 and Figure S1). To ascertain whether this phenotype was influenced by the action of AR, we treated AR-Tg mice with Sorbinil, a potent AR inhibitor [8]. The lifelong administration of Sorbinil to AR-Tg mice largely prevented the thickening of the RNFL complex routinely seen in AR-Tg untreated controls at 18 weeks of age (*p* < 0.001; Figure 2; additional images depicting measurements of RNFL complex thickness are shown in Figure S2).

**Figure 3:**
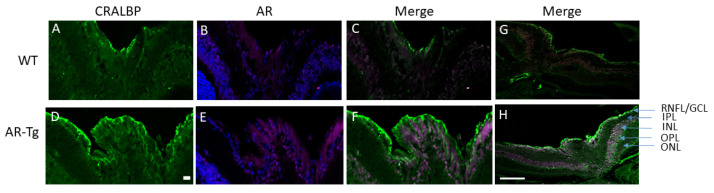
Distribution of AR and cellular retinaldehyde binding protein (CRALBP), a well-characterized marker for Müller glia in WT (**A**–**C**,**G**) and AR-Tg (**D**–**F**,**H**) mouse eyes. Cryosections of retinal tissue were immunostained for the presence of human AR (magenta) and CRALBP (green) in 24-week-old wild-type control (WT) and age-matched AR-Tg mice. Images of the AR immunostain also showed nuclei identified by DAPI-staining (blue). Panels G and H show full thickness retinal images, where strong CRALBP positivity can be seen in presumptive Müller glia in AR-Tg extending from the RNFL complex to the ONL. Scale bar = 100 µm. Images are representative of ≥ 3 animals. Labeling of retinal layers is as in Figure 1.

Given the profound effect of Sorbinil on the prevention of RNFL complex thickening in AR-Tg mice, we next sought to verify expression of human AR in the affected tissue layer. Immunofluorescence staining for human AR in cryosections of retina taken from WT and AR-Tg mice showed strong immunostaining in AR-Tg mice. The staining appeared to not be limited to a particular laminar region, but rather showed patterns that resembled columns that radiated from the outer to inner retinal layers (Figure 3). 

Müller glia (MG) are known to extend from the inner to outer retina and form foot plates at the interface of the inner retinal compartment and the retinal inner limiting membrane. To ascertain if MG populate the area of thickened cells adjacent to the inner limiting membrane observed in our AR-Tg model, we carried out immunostaining for cellular retinaldehyde binding protein (CRALBP), a protein abundantly expressed in the MG and retinal pigment epithelium [9]. As shown in Figure 3, the intense staining of CRALBP was observed in cells lining the retinal ILM in AR-Tg mice. Cells in this region of nontransgenic controls were also positive for CRALBP immunostaining, but at a lower intensity when compared with AR-Tg (Figure 3). Immunostaining for CRALBP, as in the case of AR, included areas resembling a columnar pattern terminating at the RNFL. Taken together, these results suggest that human AR expression in the AR-Tg mouse occurs in cells associated with the thickened retinal nerve fiber layer, including the Müller glia.

### 2.2. AR-Dependent Structural and Functional Changes to Retinal Ganglion Cells

**Figure 4:**
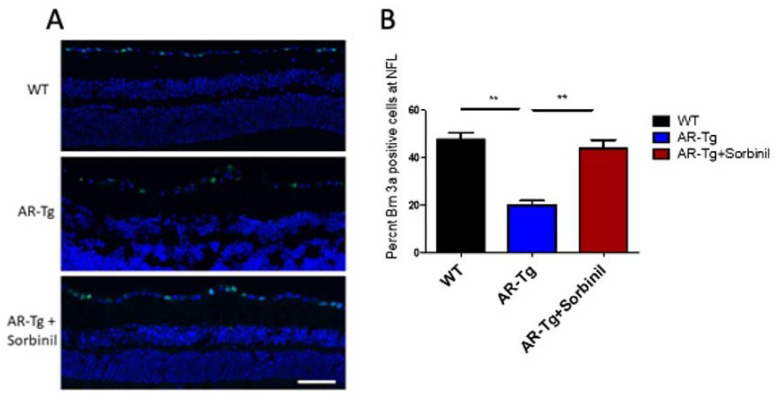
Abundance of Brn3a-positive ganglion cells. (**A**) Immunofluorescence staining for Brn3a (green) in nontransgenic control (WT) and AR-Tg mice as well as AR-Tg mice treated with Sorbinil. Mice were 24 weeks of age. Cell nuclei were visualized with DAPI staining (blue). Scale bar = 50 µm. (**B**) Quantitation of Brn3a-positive cells. Brn3a-positivity was assessed by counting ≥3 fields from ≥3 animals from each group. ** *p* < 0.01.

Axons extending from retinal ganglion cells (RGC) organize into bundles that course along the RNFL and leave the eye through the optic nerve to connect to the brain. Given the phenotype of an apparent activation of Müller glia to a proinflammatory and gliotic state, which is known to induce apoptosis of RGCs, we next determined whether such changes to Müller glia may result in a loss of RGCs in our AR-Tg model [10,11]. The number of Brn3a-positive cells, a well-characterized marker for RGCs, within the nerve fiber layer was used as a surrogate for the total number of ganglion cells in the retinas of mice at 35 weeks of age [12]. Immunostaining for Brn3a revealed a significant loss of RGCs in AR-Tg mice compared with age-matched wild-type controls, with AR-Tg mice having less than half the number of Brn3a-positive RGCs at the RNFL compared to the wild type (Figure 4A). A role for AR in the loss of Brn3a-positive RGCs can be deduced by the rescue effect of Sorbinil, which resulted in the virtually complete protection of RGCs against the deleterious effects seen in the AR-Tg mice (Figure 4B). 

**Figure 5:**
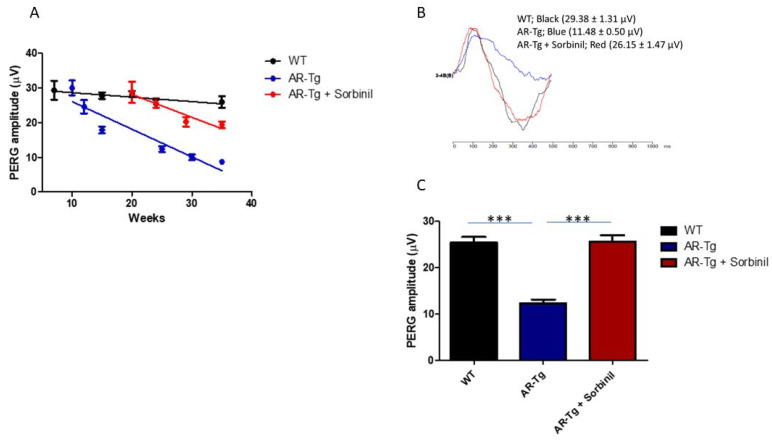
AR-Tg mice experienced an age-dependent loss of ganglion cell function that could be moderated by the inhibition of AR activity. Ganglion cell activity was determined by PERG in AR-Tg and nontransgenic control mice. (**A**) Serial PERG amplitude recordings were performed on the same animal from age 7 to 35 weeks. Nontransgenic control (WT); black line, AR-Tg; blue line, AR-Tg treated with Sorbinil; red line, error bars represent the SD (*n* = 5). (**B**) Average PERG amplitudes at age 24 weeks indicated a >60% reduction in PERG of AR-Tg animals compared to age-matched WT controls. (**C**) Treatment of AR-Tg animals with Sorbinil; however, returned PERG amplitude to near normal levels (*** *p* < 0.001). Data shown as the mean ± SEM, *n* ≥ 5 for each group, at 24 weeks. *** *p* < 0.001.

The loss of RGCs revealed in immunostaining studies was also reflected in a loss of ganglion cell function, measured by pattern electroretinography (PERG). PERG is a noninvasive method used to measure the light-evoked electrical activity of RGCs. We observed through longitudinal studies that AR-Tg mice experienced an age-dependent loss of RGC function over a period of 35 weeks. A potential role for AR in this reduction in PERG amplitude was demonstrated by a substantial delay, relative to untreated AR-Tg mice, in the loss of PERG signal activity when mice were treated with Sorbinil. AR-Tg mice showed a substantial loss of PERG amplitude compared to wild-type controls, beginning at 10 weeks of age (Figure 5). In contrast, wild-type and Sorbinil-treated AR-Tg mice showed similar PERG amplitudes up to 20 weeks of age. Thereafter, the PERG amplitudes began to decline in Sorbinil-treated mice relative to WT controls but were still much stronger than in the untreated AR-Tg mice. When measured at 24 weeks of age, the PERG “waves” of AR-Tg mice showed substantial losses compared to WT controls (Figure 5). In contrast, the PERG wave form of Sorbinil-treated mice largely overlapped that of the age-matched WT controls. 

### 2.3. AR-Dependent Increases in Markers of Retinal Inflammation

**Figure 6:**
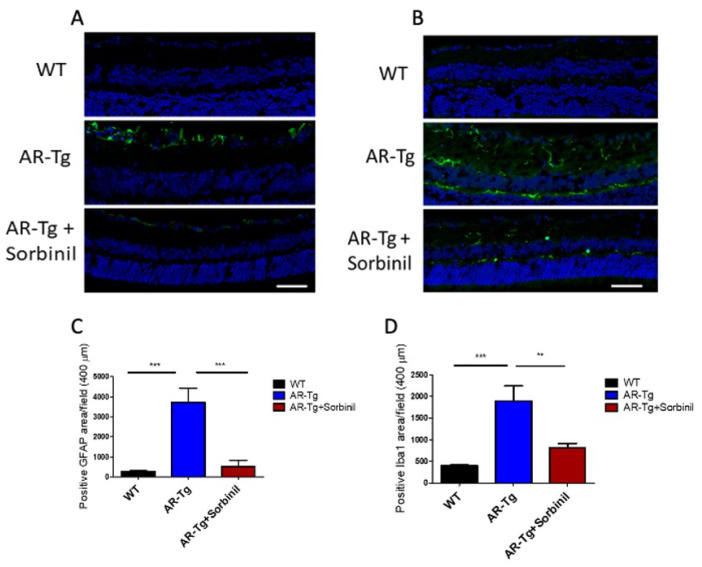
Retinas from AR-Tg animals showed an increase in expression of inflammatory markers that was reduced with Sorbinil treatment. (**A**) Analysis of immunofluorescence images showed a significantly increased number of activated glia (GFAP signal, green) in the inner nuclear layer of AR-Tg mice compared to age-matched controls. The area of GFAP signal in AR-Tg mice treated with Sorbinil was significantly reduced. (**B**) Analysis of immunofluorescence images demonstrated a significantly larger number of retinal microglia (Iba-1 signal, green) throughout the inner and outer nuclear layers of AR-Tg mice compared to age-matched WT controls. The area of Iba-1 signal in AR-Tg mice treated with Sorbinil was significantly reduced compared to untreated AR-Tg mice, but did not return to the level seen in WT animals. (**C**,**D**) GFAP and Iba-1 expression levels were quantified by pixel counts in microscopic fields in retinal sections from designated animal strains, respectively. Tissues were counter-stained with DAPI (blue). Mice were 24 weeks of age. Scale bar equals 50 µm. ** *p* < 0.01, *** *p* < 0.001.

Elevated expression of AR has been associated with a shift toward a proinflammatory phenotype characterized by increased levels of activated retinal microglia [13]. Since activated retinal Müller glia suspected in our AR-Tg mice would be expected to secrete proinflammatory cytokines and chemokines, we looked for corollary signs of inflammation, such as increased levels of activated retinal microglia and astrocytes. As shown in Figure 6, the abundance of cells extending to the outer nuclear layer and showing immunopositivity for glial fibrillary acidic protein (GFAP), a marker for activated retinal glia, was dramatically elevated in AR-Tg retina compared to the wild-type controls (*p* < 0.001). Similarly, cells staining positive for Iba-1, a marker for retinal microglia, were significantly elevated in comparison to the wild type (*p* < 0.001). The treatment of AR-Tg mice with Sorbinil largely prevented the upregulation of both of these markers of inflammation.

## 3. Discussion

Aldose reductase, the first and rate-limiting enzyme of the polyol pathway of glucose metabolism, has been implicated in the pathogenesis of diabetic complications in the eye, including retinopathy and cataracts [1]. Accelerated flux of glucose through the polyol pathway leads to a variety of metabolic imbalances, including the accumulation of sorbitol to osmotically significant levels in some tissues, the depletion of ATP, changes to ratios of NAD/NADH, and increased oxidative stress due to reduced levels of NADPH and glutathione [14,15,16,17]. Our studies and those of others with AR-transgenic mice have demonstrated that the increased lens-directed expression of AR leads to an increased incidence of cataracts after the induction of experimental diabetes or following galactose feeding, unlike nontransgenic littermates with basal levels of AR [6,14]. In the course of characterizing our AR-transgenic mice, we observed a phenotype of marked thickening of the retinal nerve fiber layer complex in AR-transgenic mice, which was unexpected since the hybrid lens crystallin gene promoter used in these studies was intended to target transgene expression to the lens [6]. When examined at 3 weeks of age, the retinas of WT and AR-Tg mice were essentially indistinguishable, with well-defined inner and outer nuclear layers, an inner plexiform layer, and a well-populated layer of retinal ganglion cells. However, by 18 weeks, despite the preservation of the major retinal laminations, AR-Tg retinas showed a marked thickening of the retinal nerve fiber layer complex that was not apparent in age-matched nontransgenic controls. 

**Figure 7:**
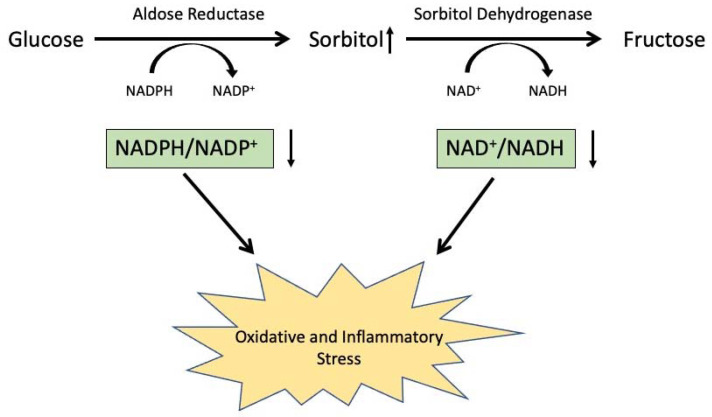
Consequences of increased AR leading to NAD(P)H redox imbalances and the induction of oxidative and inflammatory stress. Increased flux of glucose through the polyol pathway leads to sorbitol accumulation, redox changes in NAD(P)^+^/NAD(P)H ratios, and oxidative and inflammatory stress.

Several lines of evidence lead us to suspect that the activation of AR expression in Müller glia is responsible for the phenotypes we observe in our AR-Tg mouse line. The thickness of the RNFL complex in AR-Tg mice maintained on Sorbinil was essentially indistinguishable from WT, whereas AR-Tg mice not treated with the AR inhibitor showed marked thickening. Previous studies have shown that RNFL thickness is influenced by contributions from Müller cells [18], and Müller cell gliosis is elevated in diabetes [19]. Thus, it is reasonable to hypothesize that increased polyol pathway activity contributes to this phenotype and that pathway blockade with an AR inhibitor would reduce if not eliminate the severity of this phenotype, which it did. The activation of Müller glia and gliosis can result in the production of TNFα, a potent proinflammatory cytokine that causes the activation of retinal microglia and astrocytes [10], both conditions observed in our AR-Tg mice and both responsive to the attenuating effects of Sorbinil treatment. These results are consistent with previous studies demonstrating the ability of AR inhibitors to blunt the proinflammatory effects of TNFα observed in different model systems [20,21,22,23]. Figure 7 shows a schematic representing the relationships among AR and polyol pathway enzymes and the deleterious consequences of increased polyol pathway metabolism through the elevation of AR.

Diabetic retinopathy has for many years been considered a microvascular disease. However, current thinking is shifting toward a realization that the modulating influence of neuronal processes on the retinal inflammation and apoptosis of retinal ganglion cells may play an important role in the effects of diabetes in the retina [24]. While our studies were carried out in nondiabetic animals, we hypothesize that the effects of AR inhibition to prevent inflammatory activation of glial cells and astrocytes and the loss of retinal ganglion cells may be a useful strategy to prevent vision loss in the diabetic condition as well. Future studies will hopefully provide insights into these provocative ideas. 

## 4. Materials and Methods

### 4.1. Animals

The research in this study was conducted in compliance with the ARVO Statement for the Use of Animals in Ophthalmic and Vision Research, as well as an animal protocol approved by the University of Colorado Medical Center Animal Care and Use Committee (IACUC). C57BL/6J (WT control) mice were purchased from the Jackson Laboratory (Bar Harbor, ME, USA), and transgenic AR-Tg mice (homozygous for the AKR1B1 gene) were generated by our lab on a C57BL/6 background, as reported previously [6,25]. Briefly, in the AR-Tg strain of mice, the expression of the human ARK1B1 gene (AR) is under control of a hybrid of α/δ1 crystalline enhancer/promoter, leading to preferential expression in lens epithelium and fiber cells [26]. Animals of both sexes were used. All mice were housed in a humidity-controlled room under a standard 12 h light–dark cycle and fed standard chow. Where indicated, animals were treated with Sorbinil provided at a concentration of 0.25 mg/mL in drinking water. Unless indicated otherwise, Sorbinil treatment was initiated with pregnant female mice and continued in offspring drinking water for life-long treatment. We estimate that after weaning, group-housed mice received a Sorbinil dose of approximately 2–3 mg/animal/day.

### 4.2. Histology

Enucleated eyes used for the histological and immunohistochemical (IHC) staining were fixed using a modified Davidson’s fixative (1 part acetic acid, 1 part 10% formalin, 3 parts 95% ethanol, and 3 parts water) for approximately 18 h, transferred to 10% neutral buffered formalin (ThermoFisher Scientific, Waltham, MA, USA) for a further 4 h, and then embedded in paraffin. Sagittal eye sections (3.5 µm) were cut and stained with Mayer’s hematoxylin and eosin Y (H&E: Richard-Allan Scientific, Kalamazoo, MI, USA) or used for IHC analysis. Bright-field color images were obtained using a Nikon Eclipse 80i microscope (Nikon, Melville, NY, USA) equipped with a Nikon D5-Fi1 color camera, a Nikon CFI 4x or 20x/Plan Fluor objective lens, and the NIS-Elements software package.

The thickness of the RNFL complex was determined using the length measurement tool contained within the Nikon NIS-Elements software package version 5.20.02. For each image, at least six evenly spaced measurements were taken (while avoiding areas with blood vessels), representing the distance from the base of the RGC layer to the retinal inner limiting membrane. Measurements were taken over a distance of approximately 100–400 µm extending in either direction from the center of the optic nerve. 

Enucleated eyes used for immunofluorescence staining were fixed for 2 h in 4% paraformaldehyde, infused at 4 °C with an increasing concentration series (10–30%) of sucrose solutions in phosphate buffered saline (PBS), and then flash frozen in OCT (Sakura Finetech, Torrance, CA, USA). Sagittal eye sections (10 µm) were first washed in PBS to remove excess OCT and then blocked for 1 h at room temperature in PBS containing 10% bovine serum albumin (Sigma-Aldrich) and 10% normal goat serum (Gibco/ThermoFisher Scientific, Waltham, MA, USA). Tissue sections were then incubated with the primary antibody in blocking solution overnight at 4 °C and washed three times in PBS containing 0.1% Tween-20 (PBST; Sigma-Aldrich, St. Louis, MO, USA). For detection of unconjugated primary antibodies, tissue sections were incubated for 40–60 min at room temperature in the dark with the appropriate secondary antibody (Alexa488 or Alexa594) diluted in blocking solution followed by three PBST washes. All sections were then mounted under coverslips using Fluoro-Gel II (Electron Microscopy Science, Hatfield, PA, USA) containing DAPI as a nuclear stain. Immunofluorescent images were collected using a laser scanning confocal microscope (C2+; Nikon Instruments, Inc., Melville, NY, USA) equipped with a Nikon 20x/0.75 (Waltham, MA, USA) software package. Each set of experimental images was captured using similar microscope configurations, including laser intensity and brightness. At least three replicates for each set of experiments were analyzed.

The following mouse monoclonal antibodies were used at the given dilutions: anti- glial fibrillary acidic protein (GFAP; ThermoFisher Scientific, Waltham, MA, USA) diluted 1:150, anti-ionized calcium-binding adapter molecule 1 (Iba1, Wako Chemicals, Richmond, VA, USA) diluted 1:150, monoclonal anti-Brn3a conjugated with Alexa488 (Santa Cruz, TX, USA) diluted 1:75, monoclonal anti-AR conjugated with Alexa594 (Santa Cruz, TX, USA) diluted 1:100, and anti-CRALBP antibody (Abcam, Cambridge, UK) diluted 1:250. Secondary antibodies (diluted 1:500) included Alexa 488 goat anti-mouse IgG and Alexa594 goat anti-mouse IgG (Invitrogen, Waltham, MA, USA). 

Immunofluorescence analysis: The analysis of immunofluorescent images was performed using the Nikon NIS-Elements analysis software package. The percent of Brn3a-positive cells within the nerve fiber layer was determined using the “Object Count” feature. A 400 micron long region of nerve fiber layer was first isolated using the “polygon” region of interest (ROI) tool. Objects in the DAPI channel (total numbers of nuclei) and Alexa488 channel (Brn3a-positive nuclei) were identified by first eliminating background fluorescence (bottom 12–15% of the signal) using the “Thresholding” tool, setting “Smooth” option to “1x”, “Separate” option to “ON”, and restricting the size to objects greater than 2 microns. The areas of positive staining were obtained using the “Field Measurement” feature. A 400 micron by 200 micron region of the retina was first isolated using the rectangular ROI tool. After eliminating the background fluorescence (bottom 12.5% of the signal) using the “Thresholding” tool, field measurements were performed to obtain the area of positive fluorescent signal. At least three replicates per each set of experiments were analyzed.

### 4.3. Pattern Electroretinogram (PERG) Recordings

PERG recording was performed as previously described using the JORVEC instrument (Jorvec Corp., Miami, FL, USA) [27,28]. Mice were anesthetized by intraperitoneal (IP) injection of a combination of ketamine and xylazine. The recording needle electrode (Ambu Inc. Columbia, MD, USA) was placed directly under the skin of the snout, the reference needle electrode was placed under the skin between the ears of the mice, and the ground electrode was placed in the leg or at the base of the tail of the mice. Pattern stimuli was generated on two identical LED displays with black-white horizontal bars using stimulation pattern #4 (four white elements/four black elements). Other acquisition parameters settings for recording PERG responses were as per the manufacturer’s suggestions: Gain, 100k; Low pass filter, 100 Hz; High pass filter, 1 Hz; Sweeps, 372; Sampling time, 1 millisecond. 

### 4.4. Statistics

All data shown are means ± SEM. Quantifications of immunofluorescent-positive cell staining were analyzed using *t*-tests or one-way ANOVAs, followed by Tukey’s Multiple Comparison Test, in statistical software by GraphPad Prism 7.0 (GraphPad Software, Inc., La Jolla, CA, USA). * *p* value < 0.05 was considered statistically significant.

## Data Availability

The data presented in this study are available in this article.

## Supplementary Materials

The following are available online at https://www.mdpi.com/article/10.3390/metabo11070450/s1, Figure S1: Quantification of thickness of the RNFL complex in 3 weeks old mice, Figure S2: Typical histology pictures used for measurements of thickness of the RNFL complex in mice at 18 weeks of age, Figure S3: Immunostaining for GFAP in AR-Tg (24 weeks).

## Abbreviations

AR: aldose reductase; AR-Tg, aldose reductase transgenic mouse; IF, immunofluorescent; MG, Müller glia; PERG, pattern electroretinography; RGC, retinal ganglion cell; RNFL, retinal nerve fiber layer.

## References

[1] Chang K.C., Petrash J.M. (2018). Aldo-Keto Reductases: Multifunctional Proteins as Therapeutic Targets in Diabetes and Inflammatory Disease. Adv. Exp. Med. Biol..

[2] Williamson J.R., Chang K., Frangos M., Hasan K.S., Ido Y., Kawamura T., Nyengaard J.R., van den Enden M., Kilo C., Tilton R.G. (1993). Hyperglycemic pseudohypoxia and diabetic complications. Diabetes.

[3] Chang K.C., Petrash J.M. (2015). Aldose Reductase Mediates Transforming Growth Factor beta2 (TGF-beta2)-Induced Migration and Epithelial-To-Mesenchymal Transition of Lens-Derived Epithelial Cells. Investig. Ophthalmol. Vis. Sci..

[4] Chang K.C., Ponder J., Labarbera D.V., Petrash J.M. (2014). Aldose reductase inhibition prevents endotoxin-induced inflammatory responses in retinal microglia. Investig. Ophthalmol. Vis. Sci..

[5] Tammali R., Reddy A.B., Ramana K.V., Petrash J.M., Srivastava S.K. (2009). Aldose reductase deficiency in mice prevents azoxymethane-induced colonic preneoplastic aberrant crypt foci formation. Carcinogenesis.

[6] Snow A., Shieh B., Chang K.C., Pal A., Lenhart P., Ammar D., Ruzycki P., Palla S., Reddy G.B., Petrash J.M. (2015). Aldose reductase expression as a risk factor for cataract. Chem. Biol. Interact..

[7] Chang K.C., Snow A., LaBarbera D.V., Petrash J.M. (2015). Aldose reductase inhibition alleviates hyperglycemic effects on human retinal pigment epithelial cells. Chem. Biol. Interact..

[8] Nakano T., Petrash J.M. (1996). Kinetic and spectroscopic evidence for active site inhibition of human aldose reductase. Biochemistry.

[9] Saari J.C., Nawrot M., Kennedy B.N., Garwin G.G., Hurley J.B., Huang J., Possin D.E., Crabb J.W. (2001). Visual cycle impairment in cellular retinaldehyde binding protein (CRALBP) knockout mice results in delayed dark adaptation. Neuron.

[10] Tezel G. (2008). TNF-alpha signaling in glaucomatous neurodegeneration. Prog. Brain Res..

[11] Hu X., Xu M.X., Zhou H., Cheng S., Li F., Miao Y., Wang Z. (2020). Tumor necrosis factor-alpha aggravates gliosis and inflammation of activated retinal Muller cells. Biochem. Biophys. Res. Commun..

[12] Nadal-Nicolas F.M., Jimenez-Lopez M., Sobrado-Calvo P., Nieto-Lopez L., Canovas-Martinez I., Salinas-Navarro M., Vidal-Sanz M., Agudo M. (2009). Brn3a as a marker of retinal ganglion cells: Qualitative and quantitative time course studies in naive and optic nerve-injured retinas. Investig. Ophthalmol. Vis. Sci..

[13] Chang K.C., Shieh B., Petrash J.M. (2016). Aldose reductase mediates retinal microglia activation. Biochem. Biophys. Res. Commun..

[14] Lee A.Y., Chung S.K., Chung S.S. (1995). Demonstration that polyol accumulation is responsible for diabetic cataract by the use of transgenic mice expressing the aldose reductase gene in the lens. Proc. Natl. Acad. Sci. USA.

[15] Lanaspa M.A., Ishimoto T., Li N., Cicerchi C., Orlicky D.J., Ruzycki P., Rivard C., Inaba S., Roncal-Jimenez C.A., Bales E.S. (2013). Endogenous fructose production and metabolism in the liver contributes to the development of metabolic syndrome. Nat. Commun..

[16] Lee A.Y., Chung S.S. (1999). Contributions of polyol pathway to oxidative stress in diabetic cataract. FASEB J..

[17] Srivastava S.K., Yadav U.C., Reddy A.B., Saxena A., Tammali R., Shoeb M., Ansari N.H., Bhatnagar A., Petrash M.J., Srivastava S. (2011). Aldose reductase inhibition suppresses oxidative stress-induced inflammatory disorders. Chem. Biol. Interact..

[18] Frenkel S., Goshen G., Leach L., Pe’er J., Mimouni M., Blumenthal E.Z. (2018). Peripapillary distribution of Muller cells within the retinal nerve fiber layer in human eyes. Exp. Eye Res..

[19] Li H., Chen D., Sun W., Chen J., Luo C., Xu H., Ma J.H., Tang S. (2021). KATP Opener Attenuates Diabetic-Induced Muller Gliosis and Inflammation by Modulating Kir6.1 in Microglia. Investig. Ophthalmol. Vis. Sci..

[20] Ramana K.V., Friedrich B., Bhatnagar A., Srivastava S.K. (2003). Aldose reductase mediates cytotoxic signals of hyperglycemia and TNF-alpha in human lens epithelial cells. FASEB J..

[21] Chandra D., Ramana K.V., Friedrich B., Srivastava S., Bhatnagar A., Srivastava S.K. (2003). Role of aldose reductase in TNF-alpha-induced apoptosis of vascular endothelial cells. Chem. Biol. Interact..

[22] Ramana K.V., Bhatnagar A., Srivastava S.K. (2004). Inhibition of aldose reductase attenuates TNF-alpha-induced expression of adhesion molecules in endothelial cells. FASEB J..

[23] Ramana K.V., Bhatnagar A., Srivastava S.K. (2004). Aldose reductase regulates TNF-alpha-induced cell signaling and apoptosis in vascular endothelial cells. FEBS Lett..

[24] Simo R., Stitt A.W., Gardner T.W. (2018). Neurodegeneration in diabetic retinopathy: Does it really matter?. Diabetologia.

[25] Zablocki G.J., Ruzycki P.A., Overturf M.A., Palla S., Reddy G.B., Petrash J.M. (2011). Aldose reductase-mediated induction of epithelium-to-mesenchymal transition (EMT) in lens. Chem. Biol. Interact..

[26] Reneker L.W., Chen Q., Bloch A., Xie L., Schuster G., Overbeek P.A. (2004). Chick delta1-crystallin enhancer influences mouse alphaA-crystallin promoter activity in transgenic mice. Investig. Ophthalmol. Vis. Sci..

[27] Chou T.H., Bohorquez J., Toft-Nielsen J., Ozdamar O., Porciatti V. (2014). Robust mouse pattern electroretinograms derived simultaneously from each eye using a common snout electrode. Investig. Ophthalmol. Vis. Sci..

[28] Luo X., Frishman L.J. (2011). Retinal pathway origins of the pattern electroretinogram (PERG). Investig. Ophthalmol. Vis. Sci..

