# Supplementary material for: Diabetes-Independent Retinal Phenotypes in an Aldose Reductase Transgenic Mouse Model
Source: Metabolites. 2021 Jul 10;11(7):450. doi: 10.3390/metabo11070450 (PMC8305400; doi:10.3390/metabo11070450)
Supplement: Supplementary file 1 [file metabolites-11-00450-s001.zip › metabolites-1252932-supplementary.pdf]

Supplementary Materials

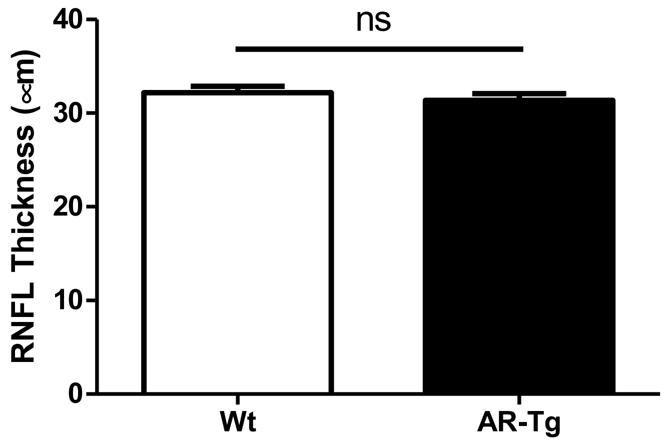

Figure S1. Quantification of thickness of the RNFL complex in 3 weeks old mice. Eyes were collected at three weeks of age and used to produced paraffin sections for H&E staining. The thickness of the retinal nerve fiber layer complex in 3 week old mice was measured at ≥5 sites located along the retina to either side of the optic nerve, similar to that for 18 week old mice shown in Figure S2.

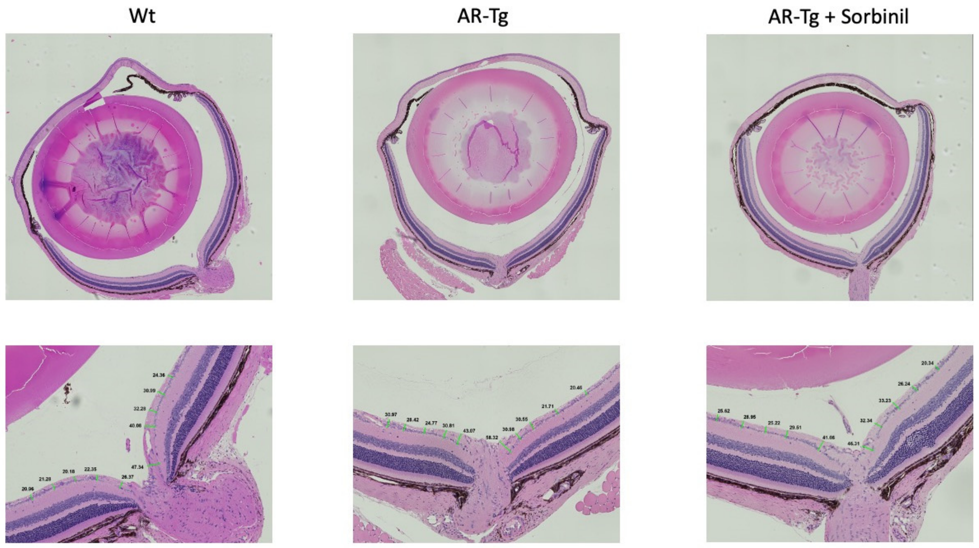

Figure S2. Typical histology pictures used for measurements of thickness of the RNFL complex in mice at 18 weeks of age. Thickness (µm) of the RNFL complex are shown by green hash marks at various sites surrounding the optic nerve.

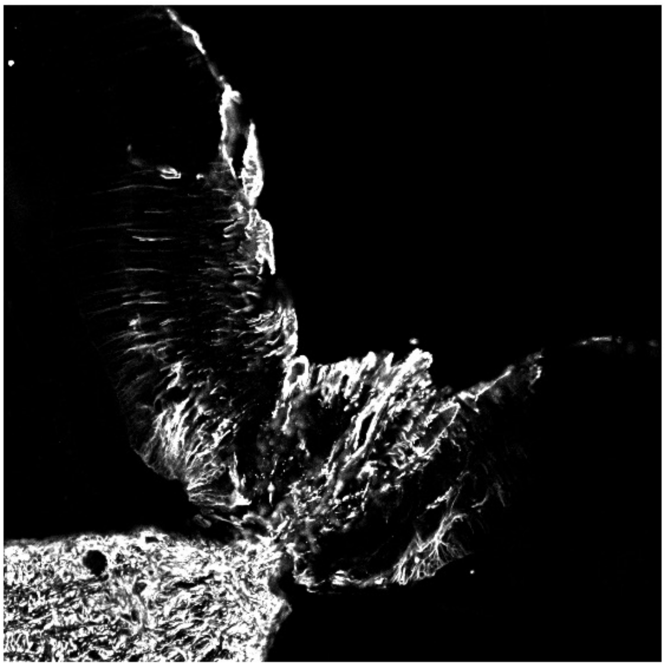

Figure S3. Immunostaining for GFAP in AR-Tg (24 weeks). Strong immunostaining was observed in outer retinal layers as well as in the RNFL complex layer.
